# Idiopathic Multicentric Castleman Disease with Cutaneous Manifestation: Case Report

**DOI:** 10.3390/medicina58091222

**Published:** 2022-09-05

**Authors:** Christoforos S. Kosmidis, Chrysi Maria Mystakidou, Georgios Koimtzis, Evanthia Papadopoulou, Vasiliki Theodorou, Nikolaos Iason Katsios, Eleni Georgakoudi, Christina Sevva, Ioannis Charalampous, Nikolaos Varsamis, Charilaos Koulouris, Christina Michael, Konstantinos Papadopoulos, Georgios Anthimidis, Sofia Baka

**Affiliations:** 1 European Interbalkan Medical Center, 10 Asklipiou Street, 55535 Pylaia, Greece; 2 3rd Surgical Department, University General Hospital of Thessaloniki “AHEPA”, School of Medicine, Faculty of Health Sciences, Aristotle University of Thessaloniki, 1st St. Kiriakidi Street, 54621 Thessaloniki, Greece; 3 Medical School, Faculty of Health Sciences, Aristotle University of Thessaloniki, 54124 Thessaloniki, Greece; 4 Cardiff Transplant Unit, University Hospital of Wales, Cardiff and Vale University Health Board, Cardiff CF14 4XW, UK; 5 Shakolas Educational Centre for Clinical Medicine, University of Cyprus, Old Road Nicosia-Lemesos 215/6, 1678 Nicosia, Cyprus; 6 Medical School, Faculty of Health Sciences, University of Ioannina, 45110 Ioannina, Greece

**Keywords:** Castleman disease, idiopathic, cutaneous manifestation, lymphoproliferative disorders, plasmacytic variant, case report

## Abstract

Castleman disease constitutes a rare class of lymphoproliferative disorders, with an estimated incidence of 21 to 25 per million patient years. The idiopathic subtype exhibits a significantly diverse clinical presentation, which can imitate many autoimmune, malignant, and infectious diseases. Cutaneous manifestations are uncommon and require in-depth investigation, especially when concurrent lymphadenopathy is present. A 79-year-old female, with a chronic, complicated erysipelas-like lesion, presented with bilaterally enlarged inguinal lymph nodes; after surgical excision, their histopathological examination revealed Castleman disease. Even though it is a benign condition, patients are often predisposed to developing certain types of malignancies, which can deteriorate their prognosis. An accurate and early diagnosis, along with effective treatment and prevention of recurrence, is of utmost importance in order to increase the patients’ overall survival and quality of life.

## 1. Introduction

Castleman disease (CD) was first introduced in 1956, when Dr. Benjamin Castleman published several case reports of a single enlarged lymph node, most commonly in the mediastinum, which showcased distinct vascularity and hyalinization [1].

Almost 70 years later, CD remains a rare and poorly understood entity, encompassing a variety of lymphoproliferative disorders, presenting with lymphadenopathy and sharing characteristic histologic features. It is typically divided into unicentric (UCD), with a solitary affected lymph node, and multicentric (MCD), engaging at least two lymph node stations. Interleukin-6 (IL-6) appears to have a central part in MCD’s pathogenesis [2]. The idiopathic subtype of MCD (iMCD) has an estimated incidence of 5 per million patient years (PYs) [3,4]; it constitutes approximately 1/3 of all MCD cases and exhibits a wide range of signs and symptoms, along with an equally diverse clinical course [5].

Cutaneous involvement is scarcely reported in the literature; the skin lesions can present concurrently with constitutional symptoms, yet, occasionally, they may precede any other indication of CD [6]. Herein, we present a case of iMCD in an elderly woman, with a cutaneous initial manifestation.

## 2. Case Presentation

**Figure 1:**
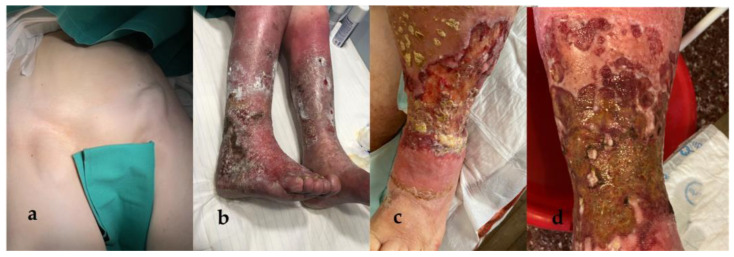
Preoperative images of the patient’s enlarged inguinal lymph nodes (**a**) and the chronic lesion of the lower extremities; image from an extensive archive, similar to that of the lesion at the time of presentation (**b**). The lesions’ clinical course was complicated by ulcerations, with active infection and crusting (**c**,**d**).

A 79-year-old female presented with bilaterally enlarged inguinal lymph nodes, reporting that they had grown substantially during last month. Upon physical examination, the lymph nodes were of hard consistency, with a prominent abnormal shape, mobile, and sensitive to the touch (Figure 1a).

The patient was also suffering from a chronic, bilateral erysipelas-like erythema of the lower extremities, which had first appeared 7 years ago (Figure 1b). It was of unknown etiology and exhibited an unfavorable clinical course, with the progressive development of multiple, extensive, ulcerative lesions (Figure 1c,d). The patient had been hospitalized numerous times in the past for local, recurrent bacterial and fungal infections, often with resistant strains of *Pseudomonas aeruginosa*, *Proteus mirabilis,* and *Klebsiella pneumoniae*, and in need of surgical debridement. At the time of presentation, she was under medication with dalbavancin, after a tissue culture of the affected area had revealed Gram (+) infection.

Chronic concomitant findings included bilateral lymphedema of the lower extremities, subcutaneous edema, slightly enlarged pelvic and inguinal lymph nodes, and elevated serum C-reactive protein (CRP) (15.5 mg/dL, normal values (nv) are <1.0 mg/dL), all of which had been deemed inflammatory and attributed to the patient’s persistent cutaneous infections. Additionally, hemoglobin levels (Hb) were slightly decreased (10 mg/dL, normal range 11.5–16.5 mg/dL), while her white blood cell (WBC) count was within the normal range (4.0–11.0 K/μL). Lab values regarding renal function and liver enzymes were also normal; serum creatinine was 0.8 mg/dL (nv 0.5–0.9 mg/dL), serum urea was 44 mg/dL (nv 10–50 mg/dL), serum glutamic-oxaloacetic transaminase (SGOT) was 24 U/L (nv 10–35 U/L), serum glutamic pyruvic transaminase (SGPT) was 11 U/L (nv 10–35 U/L), γ-glutamyl transpeptidase (γ-GT) was 10 U/L (nv 5–36 U/L), and alkaline phosphatase (ALP) was 96 U/L (nv 35–104 U/L). Lactate dehydrogenase (LDH) was found to be approximately 311 U/L (nv 240–470 U/L).

Laboratory tests for cancer markers, such as carcinoembryonic antigen (CEA) and CA 19.9, were negative, as were tests for autoantibodies, such as antinuclear antibodies (ANA) and antineutrophil cytoplasmic antibodies (ANCA). Previous CT imaging had revealed hepatomegaly, with an overall craniocaudal diameter of 18 cm.

**Figure 2:**
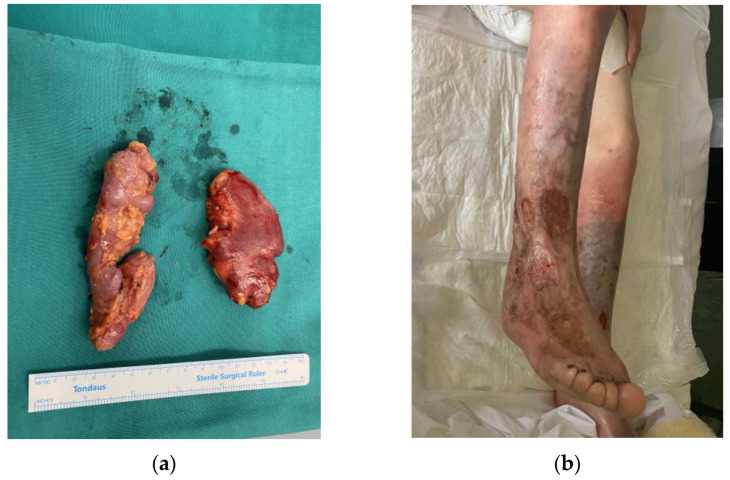
The surgical specimens of the enlarged inguinal lymph nodes (**a**). The patient’s cutaneous lesion one year after treatment (**b**).

Owing to the lymph node’s location and significant increase in size, the patient had trouble performing daily activities, such as walking; therefore, a total surgical excision of both lymph nodes was decided. Under local anesthesia and monitored anesthesia care (sedation), the patient underwent a total resection of both enlarged lymph nodes. The surgical specimens of the left and right enlarged inguinal lymph node measured approximately 8.2 cm × 4 cm and 10.5 cm × 4 cm, respectively (Figure 2a).

The surgical specimens were subsequently sent for histopathological examination; the lymph nodes maintained their normal architecture, while showcasing extensive hyperplasia, with mainly normal and, sporadically, indistinguishable germinal centers. Lymphocytic markers CD20 and CD3 indicated a normal distribution of B- and T-lymphocytes. Marked CD23 positivity was observed in the germinal centers’ reticular cells and in some mantle zone cells, while BCL6 positivity was limited to the germinal centers. Immunohistochemical staining for CD138 and multiple myeloma 1 protein (MUM1) revealed the presence of ample plasma cells; the observed plasmacytes exhibited polyclonal κ- and λ- light chains. Testing with BCL2, D1, CD15, and CD30 turned out negative; no Reed–Sternberg cells were observed. Proliferation marker ki-67 was increased (30%), primarily in the germinal centers. In situ detection for Epstein-Barr virus (EBV) was negative.

**Figure 3:**
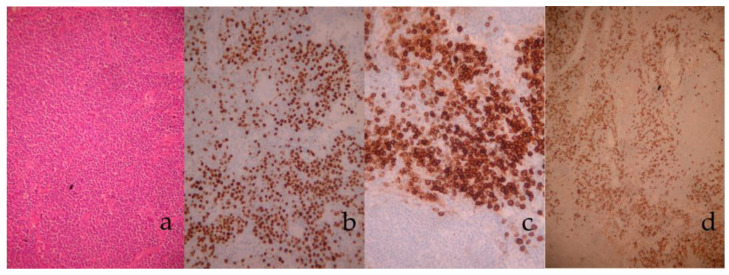
Histopathological and immunohistochemistry images: extensive plasma cell-like infiltration H&E × 100 (**a**); positive staining for MUM1 × 100 (**b**) and CD138 × 200 (**c**); κ- light chains × 100 (**d**).

The aforementioned histopathological findings were compatible with the plasmacytic variant of CD; no additional results of diversion towards lymphoma were observed (Figure 3).

In order to determine the extent of the disease and the specific subtype of CD, an overall assessment of the patient’s clinical, laboratory, and imaging findings was performed. The patient had a brain, chest, and abdominal CT, which additionally revealed slightly enlarged pelvic, retroperitoneal, and mesenteric lymph nodes. Testing for Human Herpesvirus-8 (HHV-8) and Human Immunodeficiency virus (HIV) was also performed for the purpose of sub-classifying the patient; the results were negative.

One month post-operatively, the patient underwent six cycles of immunotherapy with siltuximab, an anti-IL6 monoclonal antibody (mAb). The patient responded well to the administered medication and, after completing her treatment, a follow-up CT was ordered; the new scan showed no signs of residual or novel enlarged lymph nodes. The chronic cutaneous lesion exhibited a significant initial improvement, yet continues to remit and relapse periodically owing to recurrent infections, despite aggressive treatment with medication and surgical interventions to prevent them (Figure 2b).

## 3. Discussion

Castleman disease, previously known as angiofollicular/giant lymph node hyperplasia, can affect people of all ages. However, the median age for a UCD diagnosis tends to be lower in comparison with that for MCD, with the latter one estimated at around 50 years of age [3,4]. A discrepancy regarding CD’s gender prevalence has also been observed; a systematic review of 128 iMCD cases revealed that MCD exhibits a male predominance, while UCD tends to affect both genders evenly [5,7]. A recent retrospective study of 273 patients further supports this case [8].

Multricentric CD can etiologically derive from a plasma cell (PC) neoplasm (POEMS-MCD, from the homonym concurrent syndrome: polyneuropathy, organomegaly, endocrinopathy, monoclonal gammopathy, and skin changes), viral infection with HHV-8 (HHV-8-MCD) or be idiopathic (iMCD) [2]. The latter subtype can be further classified, based on clinical manifestation, into iMCD-TAFRO (thrombocytopenia, ascites, reticulin fibrosis, renal dysfunction, and organomegaly) and iMCD-NOS (not otherwise specified) [9]. Our patient was classified as a case of iMCD, after testing negative for an HHV8 and HIV infection, which can often be concurrent [2,4]. Despite having significant hepatomegaly, a finding in accordance with an iMCD-TAFRO diagnosis, the patient did not meet any other criteria for TAFRO, having an overall mild clinical presentation. Therefore, she was further classified as an iMCD-NOS case.

In regard to its pathogenesis, there is a clear difference between UCD and MCD. Studies have shown that UCD most likely occurs from mutations of the platelet growth factor receptor β (PDGR β) in CD45- cells, resulting in dendritic cell proliferation [2,3,4]. The basis of MCD’s pathogenesis, however, remains unclear to this day, albeit many studies are being conducted. In the most prevalent of theories, MCD’s manifestation is attributed to intrinsic processes resulting in elevated IL-6, which consequently triggers a cytokinetic inflammatory response [2,4,10]. The PC neoplasm, in POEMS-MCD and the viral equivalent of IL-6, in HHV-8-MCD, could be the instigators of the aforementioned mechanism. This notion is strongly supported by the efficacy of IL-6 inhibition in patients with those subtypes of CD; however, iMCD stands to dispute this theory. Many patients with iMCD do not respond to IL-6 inhibition, whereas many do not exhibit increased IL-6 levels at all [4,5,10]. Other possible concepts, including a genetic background equivalent to that of UCD, or a viral etiology, have been proposed, without enough supporting evidence [4,10].

Castleman disease can manifest with a variety of clinical signs and symptoms. UCD usually presents exclusively with localized lymphadenopathy; a single enlarged lymph node in an otherwise asymptomatic individual. Commonly affected areas include the mediastinum, the axillary and inguinal regions, as well as the abdominal cavity [8]. Case reports have also highlighted extranodal incidents of CD; namely, in the pancreas, the lacrimal glands, and intramuscularly [7,11,12,13]. Constitutional symptoms in UCD are rare and, when present, give rise to suspicions of other clinical entities, rather than CD [4].

On the contrary, MCD, while presenting with prominent lymphadenopathy, usually causes additional nonspecific, yet alarming, systematic symptoms; weight loss, night sweats with fever, and anasarca edema are among the most common, prompting the patient to seek medical care. Other findings include organomegaly, mainly hepato/splenomegaly, renal dysfunction, and respiratory complications. These individuals tend to have increased inflammatory markers, particularly serum CRP, along with anemia, thrombocytopenia, and characteristic hypergammaglobulinemia [2,4,5,8,14]. POEMS and TAFRO constitute the most severe forms of MCD and, if left untreated, can lead to life-threatening multiple organ dysfunction syndrome (MODS) [7]. In rare instances, a patient with MCD can present with a mild phenotype of the disease; a large systematic review, conducted by Oksenhedler et al. [8], reported that some patients exhibit an almost asymptomatic clinical course, despite having multiple affected lymph nodes, yet in the same station.

Cutaneous manifestations of CD are an additionally intriguing entity, although rare and, usually, in association with POEMS-MCD [2,15,16]. However, various case reports of patients with other types of MCD experiencing concurrent skin disorders have emerged, indicating a potential correlation [6,17,18,19]. Recorded cutaneous manifestations in the current literature include, but are not limited to, purpura, erysipelas, papules, angiomas, telangiectasias, and pemphigus [6,17,18,19,20]. In some instances, these manifestations could precede any other indication of the disease [6,21]. It has also been suggested that such lesions could be the causative factors of CD, rather than part of its symptomatology; however, cutaneous manifestations can recess in response to treatment for CD, without requiring additional treatment or other interventions [21,22].

In our patient’s case, while her overall clinical presentation has improved after treatment with anti-IL6 mAb, the skin disorder has persisted; despite exhibiting an initial promising response to treatment, full remission has not been achieved. Nonetheless, it is possible that the lesions’ initial presentation was indeed within the spectrum of CD’s cutaneous manifestations. Recurring, concurrent bacterial and fungal infections with resistant strains, however, could have potentially altered its clinical course; irreparable damage to the skin could render it treatment-refractory, despite the underlying CD having receded.

Paraneoplastic pemphigus (PNP) can often co-exist with CD, which produced a challenge in the differential diagnosis of our patient’s cutaneous lesion, after pathology results established the diagnosis of CD. However, the patient did not exhibit mucosal involvement, which constitutes the most typical and consistent presentation of PNP, especially in the oral cavity. Lack of ocular and pulmonary involvement in the patient’s symptomatology was also indicative of a correlation with CD, rather than PNP. Moreover, cutaneous lesions in PNP tend to be generalized, contrary to our patient’s clinical presentation.

Another important aspect to consider, regarding both CD’s clinical manifestation and prognosis, is the affected individuals’ predisposition to developing certain types of malignancies. This phenomenon has mostly been observed in HHV-8-MCD; regardless of an HIV co-infection, these patients exhibit a tendency for developing lymphoma, either Hodgkin (HL) or non-Hodgkin (NHL), myeloma and Kaposi sarcoma [4,15,23]. However, age-matched controls have also revealed an increased prevalence of the aforementioned malignancies in patients with iMCD [5,24].

As iMCD exhibits remarkably diverse and multisystematic manifestations, its differential diagnosis presents a challenge for physicians. Imaging findings tend to be nonspecific; an affected lymph node in a CT scan can imitate both benign and malignant tumors, including lymphomas, nerve sheath tumors, or metastatic lymphadenopathy, in patients with a primary cancerous tumor [25,26]. ^18^F-Fluorodeoxyglucose positron emission tomography-CT (F-FDG PET-CT) is more diagnostic for CD. However, MCD exhibits a variable and increased standardized uptake value (SUV); therefore, this method cannot accurately differentiate CD from lymphoma [26,27]. Laboratory testing for EBV, HIV, and specific auto-antibodies can aid in excluding infectious and autoimmune diseases, such as systemic lupus erythematosus (SLE), rheumatoid arthritis, or IgG4-related disorders, and limit CDs’ extensive differential diagnosis [2,4]. However, there is no established, imaging or laboratory, test diagnostic for CD.

This predicament produced the need for a consensus regarding iMCDs’ diagnostic criteria; such an accord was published in 2017 [28]. Each patient should meet both major and at least two minor criteria; clinical and laboratory findings are considered minor pre-requisites, while major conditions include diffuse lymphadenopathy, pertaining to at least two different stations, with histopathologic findings in accordance with CD. Excisional lymph node biopsy is the gold-standard approach for providing accurate results, ensuring that the entirety of the surgical specimen is examined; an incisional biopsy or fine needle biopsy (FNB) can often yield false negative results [4,7,28].

In addition to its aforementioned diverse clinical presentation, CD’s histologic features also vary; one end of the spectrum is the hypervascular variant (HV), and on the other is the plasmacytic variant (PC). The former resembles the microscopic findings that Castleman originally described in 1956, and is most commonly observed in UCD specimens. In 1972, the PC type was identified as a distinguishable histologic variation; characterized by prominent germinal center hyperplasia and plasma cell compounds within the lymph node, it is a histologic pattern often consistent with MCD, especially HHV-8-MCD. Notably, a combination of both HV and PC qualities can co-exist in a single specimen; an entity referred to as “mixed histopathology” [2,4,9,15,23,29].

Treatment options vary according to the patients’ CD subtype, as well as their clinical presentation. For UCD, surgical excision of the affected lymph node is the first line of treatment and is almost always curative, under the condition that the mass is resectable. Recurrence of UCD after complete surgical excision, while uncommon, may require additional treatments, such as immunotherapy with anti-IL6 mAb, radiotherapy, or embolization [2,4,30].

Multicentric CDs’ treatment varies greatly according to the patients’ specific subtype. HHV-8-MCD is highly responsive to treatment with rituximab; additional use of etoposide or liposomal doxorubicin may be necessary in the case of serious symptomatology or concurrent Kaposi sarcoma, respectively [2,4]. Treatments for POEMS-MCD include chemotherapy, radiation, and autologous stem cell transplant [2,4,16].

According to the International Treatment Consensus for iMCD, its management is heavily dependent upon the severity of the disease. For non-severe cases, anti-IL6 mAb therapy constitutes the first line of treatment; siltuximab is the recommended medication for iMCD, although tocilizumab may alternatively be used. However, a considerable percentage of patients exhibit an anti-IL6-refractory type of iMCD, in which case rituximab, an anti-CD20 mAb, can be used. Corticosteroids can be implemented in any stage of the above therapeutic regimen in order to manage symptoms. In patients unresponsive to all of the above, immunomodulators, such as anakinra, thalidomide, bortezomib, and interferon-a (IFN-a), can be integrated [2,4,31].

Owing to chemotherapy’s cytotoxic repercussions, guidelines for patients with severe iMCD suggest initial treatment with siltuximab and a corticosteroid agent. However, because of the increased risk for MODS through a cytokine storm, aggressive chemotherapy is recommended upon the treatments’ failure; chemotherapy regimens administered for hematologic malignancies, such as lymphoma and myeloma, have proven effective in managing severe iMCD [2,4,31]. If those chemotherapeutic agents fail, autologous stem cell transplant has also been attempted as the last resort, with variable outcomes [31].

Patients with UCD tend to have a normal life expectancy; local recurrence and malignant diversion are rare [30]. A large retrospective study of patients with UCD, who underwent resective surgery, conducted by Talat et al., calculated an overall survival (OS) greater than 90% during a 10-year follow-up period; notably, patients with peripheral lymphadenopathy and increased lymph node resectabilty showcased optimum overall survival [30,32]. The OS of patients with MCD varies according to their specific subtype and response to treatment. Liu et al., in a systematic review of individuals with iMCD, estimated the 2-year survival at approximately 90% [5,31]. Comorbidities, TAFRO syndrome, and unresponsiveness to the administered medication have a negative effect on the patients’ prognosis, resulting in the evident discrepancy between the life expectancy of patients with UCD and iMCD.

## 4. Conclusions

Idiopathic MCD is a rare and challenging medical entity, with a significantly diverse clinical presentation and extensive differential diagnosis. Lymphadenopathy succeeding cutaneous lesions of unknown etiology should raise clinical suspicion, as it could indicate an underlying CD, rather than being merely inflammatory.

Excisional lymph node biopsy constitutes the gold standard for an accurate diagnosis; consequent laboratory and imaging findings will sub-classify the patient and aid in selecting the appropriate treatment regimen. Owing to an increased risk of recurrence and diversion towards lymphoma, it is imperative that patients with iMCD are closely monitored, even after achieving an initial remission of their symptoms.
